# Assimilating Seizure Dynamics

**DOI:** 10.1371/journal.pcbi.1000776

**Published:** 2010-05-06

**Authors:** Ghanim Ullah, Steven J. Schiff

**Affiliations:** 1 Center for Neural Engineering, Department of Engineering Science and Mechanics, The Pennsylvania State University, University Park, Pennsylvania, United States of America; 2 Departments of Neurosurgery and Physics, The Pennsylvania State University, University Park, Pennsylvania, United States of America; University College London, United Kingdom

## Abstract

Observability of a dynamical system requires an understanding of its state—the collective values of its variables. However, existing techniques are too limited to measure all but a small fraction of the physical variables and parameters of neuronal networks. We constructed models of the biophysical properties of neuronal membrane, synaptic, and microenvironment dynamics, and incorporated them into a model-based predictor-controller framework from modern control theory. We demonstrate that it is now possible to meaningfully estimate the dynamics of small neuronal networks using as few as a single measured variable. Specifically, we assimilate noisy membrane potential measurements from individual hippocampal neurons to reconstruct the dynamics of networks of these cells, their extracellular microenvironment, and the activities of different neuronal types during seizures. We use reconstruction to account for unmeasured parts of the neuronal system, relating micro-domain metabolic processes to cellular excitability, and validate the reconstruction of cellular dynamical interactions against actual measurements. Data assimilation, the fusing of measurement with computational models, has significant potential to improve the way we observe and understand brain dynamics.

## Author Summary

To understand a complex system such as the weather or the brain, one needs an exhaustive detailing of the system variables and parameters. But such systems are vastly undersampled from existing technology. The alternative is to employ realistic computational models of the system dynamics to reconstruct the unobserved features. This model based state estimation is referred to as data assimilation. Modern robotics use data assimilation as the recursive predictive strategy that underlies the autonomous control performance of aerospace and terrestrial applications. We here adapt such data assimilation techniques to a computational model of the interplay of excitatory and inhibitory neurons during epileptic seizures. We show that incorporating lower scale metabolic models of potassium dynamics is essential for accuracy. We apply our strategy using data from simultaneous dual intracellular impalements of inhibitory and excitatory neurons. Our findings are, to our knowledge, the first validation of such data assimilation in neuronal dynamics.

## Introduction

A universal dilemma in understanding the brain is that it is complex, multiscale, nonlinear in space and time, and we never have more than partial experimental access to its dynamics. To better understand its function one not only needs to encompass the complexity and nonlinearity, but also estimate the unmeasured variables and parameters of brain dynamics. A parallel comparison can be drawn in weather forecasting [1], although atmospheric dynamics are arguably less complex and less nonlinear. Fortunately, the meteorological community has overcome some of these issues by using model based predictor-controller frameworks whose development derived from computational robotics requirements of aerospace programs in 1960s [2], [3]. A predictor-controller system employs a computational model to observe a dynamical system (e.g. weather), assimilate data through what may be relatively sparse sensors, and reconstruct and estimate the remainder of the unmeasured variables and parameters in light of available data. The result of future measured system dynamics is compared with the model predicted outcome, the expected errors within the model are updated and corrected, and the process repeats iteratively. For this recursive initial value problem to be meaningful one needs computational models of high fidelity to the dynamics of the natural systems, and explicit modeling of the uncertainties within the model and measurements [3]–[5].

The most prominent of the model based predictor-controller strategies is the Kalman filter (KF) [2]. In its original form, the KF solves a linear system. In situations of mild nonlinearity, the extended forms of the KF were used where the system equations could be linearized without losing too much of the qualitative nature of the system. Such linearization schemes are not suitable for neuronal systems with nonlinearities of the scale of action potential spike generation. With the advent of efficient nonlinear techniques in the 1990s such as the ensemble Kalman filter [6], [7] and the unscented Kalman filter (UKF) [8], [9], along with improved computational models for the dynamics of neuronal systems (incorporating synaptic inputs, cell types, and dynamic microenvironment) [10], the prospects for biophysically based ensemble filtering from neuronal systems are now strong. The general framework of the UKF differs from the extended KF in that it integrates the fundamental nonlinear models directly, along with iterating the error and noise expectations through these nonlinear equations. Instead of linearizing the system equations, UKF performs the prediction and update steps on an ensemble of potential system states. This ensemble gives a finite sampling representation of the probability distribution function of the system state [3], [11]–[15].

Our hypothesis is that seizures arise from a complex nonlinear interaction between specific excitatory and inhibitory neuronal sub-types [16]. The dynamics and excitability of such networks are further complicated by the fact that a variety of metabolic processes govern the excitability of those neuronal networks (such as potassium concentration (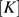) gradients and local oxygen availability), and these metabolic variables are not directly measurable using electrical potential measurements. Indeed, it is becoming increasingly apparent that electricity is not enough to describe a wide variety of neuronal phenomena. Several seizure prediction algorithms, based only on EEG signals, have achieved reasonable accuracy when applied to static time-series [17]–[19]. However, many techniques are hindered by high false positive rates, which render them unsuitable for clinical use. We presume that there are aspects of the dynamics of seizure onset and pre-seizure states that are not captured in current models when applied in real-time. In light of the dynamic nature of epilepsy, an approach that incorporates the time evolution of the underlying system for seizure prediction is required. As one cannot see much of an anticipatory signature in EEG dynamics prior to seizures, the same can be said of a variety of oscillatory transient phenomena in the nervous system ranging from up states [20], spinal cord burst firing [21], cortical oscillatory waves [22], in addition to animal [23] and human [24] epileptic seizures. All of these phenomena share the properties that they are episodic, oscillatory, and have apparent refractory periods following which small stimuli can both start and stop such events.

It has recently been shown that the interrelated dynamics of 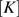 and sodium concentration (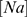) affect the excitability of neurons, help determine the occurrence of seizures, and affect the stability of persistent states of neuronal activity [10], [25]. Competition between intrinsic neuronal ion currents, sodium-potassium pumps, glia, and diffusion can produce slow and large-amplitude oscillations in ion concentrations similar to what is observed physiologically in seizures [26], [27].

Brain dynamics emerge from within a system of apparently unique complexity among the natural systems we observe. Even as multivariable sensing technology steadily improves, the near infinite dimensionality of the complex spatial extent of brain networks will require reconstruction through modeling. Since at present, our technical capabilities restrict us to only one or two variables at a restricted number of sites (such as voltage or calcium), computational models become the “lens” through which we must consider viewing all brain measurements [28]. In what follows, we will show the potential power of fusing physiological measurements with computational models. We will use reconstruction to account for unmeasured parts of the neuronal system, relating micro-domain metabolic processes to cellular excitability, and validating cellular dynamical reconstruction against actual measurements.

## Results

**Figure 1:**
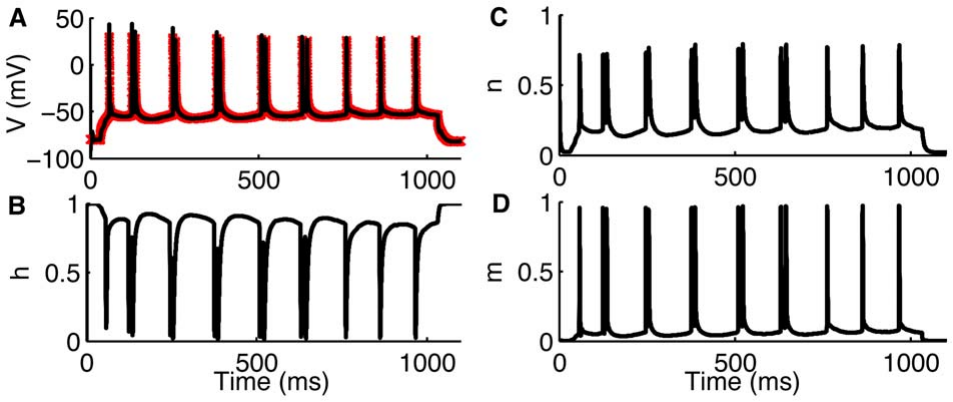
Assimilating an intracellular membrane potential recording from CA1 hippocampal pyramidal neurons. In (A) we show measured (red) and estimated (black) voltage, 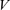. (B–D) Tracked Hodgkin-Huxley gating variables 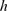, 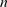, and 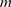 respectively. Spiking in the pyramidal cell is generated by injecting a small current of 100 picoampere for 1sec. Data provided by Jokubas Ziburkus.

**Figure 2:**
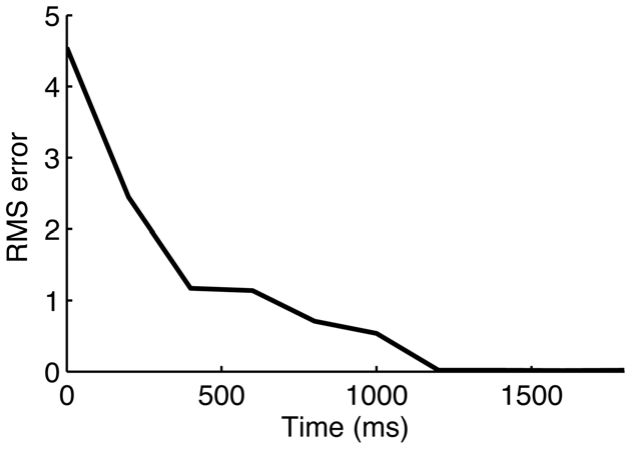
Convergence of assimilation. Root mean squared error for measured and estimated 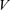 in Figure 1.

As a first example of assimilating neural data we used intracellular voltage data from a spiking pyramidal cell (PC) from the Cornu Ammonis region 1 (CA1) of rat hippocampus. Using only the noisy membrane potential measurement, 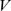, we employed modified Hodgkin-Huxley equations to reconstruct and track all of the gating variables of the ion channels: sodium channel activation and inactivation variables 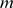 and 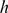, and potassium channel activation variable 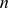 (Figure 1). Beginning with arbitrary initial conditions the root mean square (RMS) error between measured and estimated membrane potential changes with time (Figure 2). As is clear from the figure the RMS error converges to near zero within a few hundred milliseconds for the simulations shown in Figure 1. We also tracked the maximum conductance parameters of the ion channels (not shown).

### Model Inadequacy

**Figure 3:**
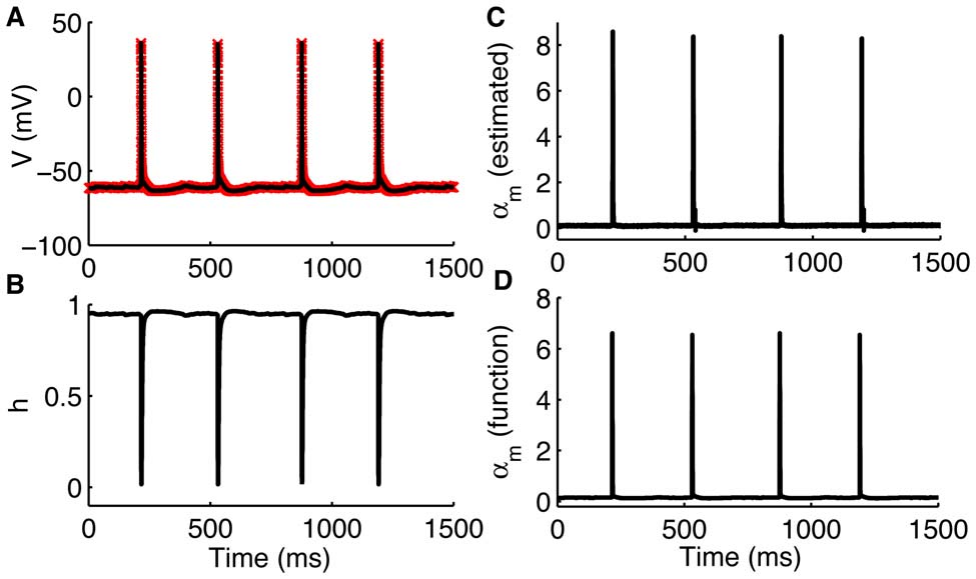
Robust neuronal dynamics tracking in the face of moderate degree of model inaccuracy. (A) measured (red), and estimated (black) voltage, 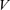, using crippled model where the critical voltage-dependent sodium rate constant, 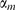, is replaced by a constant. The filter is still able to successfully estimate the gating variables (only 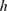 shown in (B)). 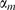 tracked as a parameter is shown in (C), while the actual functional form of 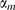 is shown in (D). Spiking in the experimentally observed cell is generated by injecting a constant current of 100 picoampere. By itself, this model cannot spike. Fused with data and allowing the parameter 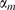 to float, it tracks 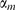 within 25% of its proper value. Data provided by Jokubas Ziburkus.

Model inadequacy is an issue of intense research in the data assimilation community – no model does exactly what nature does. To deal with inadequate models, researchers in areas such as meteorology have developed various strategies to account for the inaccuracies in the models for weather forecasting [4], [5], [29]. In complex systems such as neuronal networks, the need to account for model inadequacy is critical. To demonstrate that UKF can track neuronal dynamics in the face of moderate inadequacy, we impaired our model by setting the sodium current rate constant 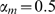 instead of using the actual complex function of 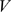, 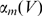 (see equation (2) for the functional form of 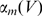), and tracked it as a parameter (Figure 3). That is, we deleted the relevant function for 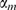 from the model and allowed UKF to update it as a parameter. The model with fixed 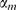 is by itself unable to spike, but when it is allowed to float when voltage is assimilated through UKF using the data from hippocampal pyramidal cells (PCs), it is capable of tracking the dynamics of the cell reasonably well. The 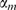 tracked by the filter is sufficiently close to its functional form values (within 25%) so that spiking dynamics can be reconstructed (Figure 3C and 3D). This occurs because Kalman filtering constantly estimates the trade off between model accuracy and measurements, expressed in the filter gain function [2], [3]. This is an excellent demonstration of the robustness of this framework. Looking at the estimated values of 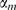 it also becomes clear that 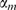 in fact should be assigned the functional form rather than a constant value.

### Tracking Neuronal Microenvironment during Seizures

**Figure 4:**
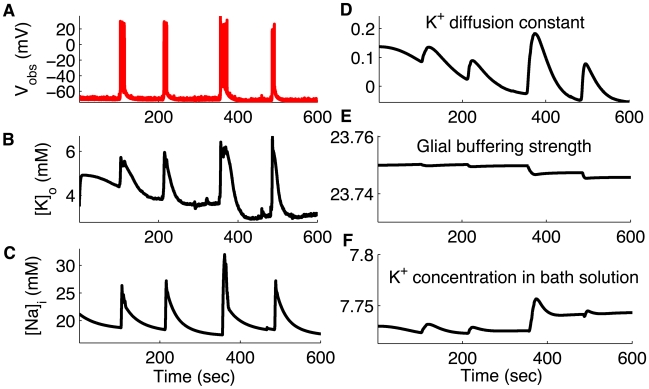
Assimilating spontaneous seizure data by whole cell recording from CA1 hippocampal pyramidal neurons. (A) Measured 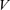 (red) from single PCs during spontaneous seizures. Estimated (black) 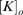 (B), 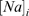 (C), 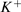 diffusion constant (D), glial buffering strength (E), and 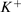 concentration in bath solution (F). Data provided by Jokubas Ziburkus. Panel (A) modified from [23] with permission American Physiological Society.

Despite decades of effort neuroscientists lack a unifying dynamical principle for epilepsy. An incomplete knowledge of the neural interactions during seizures makes the quest for unifying principles especially difficult [30]. Here we show that UKF can be employed to track experimentally inaccessible neuronal dynamics during seizures. Specifically, we used UKF to assimilate data from pairs of simultaneously impaled pyramidal cells and oriens-lacunosum moleculare (OLM) interneurons (INs) in the CA1 area of the hippocampus [23]. We then used biophysical ionic models to estimate extra- and intracellular potassium, sodium, and calcium ion concentrations and various parameters controlling their dynamics during seizures (Figure 4). In Figure 4A we show an intracellular recording from a pyramidal cell during seizures, and plot the estimated extracellular potassium concentration (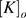) in Figure 4B. As is clear from the figure the extracellular potassium concentration oscillates as the cell goes into and out of seizures. The potassium concentration begins to rise as the cell enters seizures and peaks with the maximal firing frequency, followed by decreasing potassium concentration as the firing rate decreases and the seizure terminates. Higher 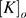 makes the PC more excitable by raising the reversal potential for 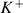 currents (equation 7). The increased 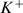 reversal potential causes the cell to burst-fire spontaneously. Whether the increased 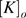 causes the cells to seize or 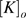 is the result of seizures has been an old question [31] whose resolution will likely take place from better understanding of the coupled 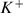 dynamics. For present purposes, it is known that increased 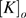 in experiments can support the generation, and increase the frequency and propagation velocity of seizures [32], [33]. Changes in the concentration of intracellular sodium ions, 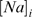, are closely coupled with the changes of 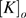 (Figure 4C). As shown in panels (4D–F) we reconstructed the parameters controlling the microenvironment of the cell. These parameters included the diffusion constant of 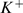 in the extracellular space, 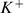 buffering strength of glia, and 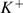 concentration in the reservoir of the perfusing solution *in vitro* (or in the vasculature *in vivo*) during seizures. Note that the ionic concentration in the distant reservoir is different from the more rapid dynamics within the smaller connecting extracellular space near single cell where excitability is determined. We were also able to track other variables and parameters such as extracellular calcium concentration and ion channel conductances.

**Figure 5:**
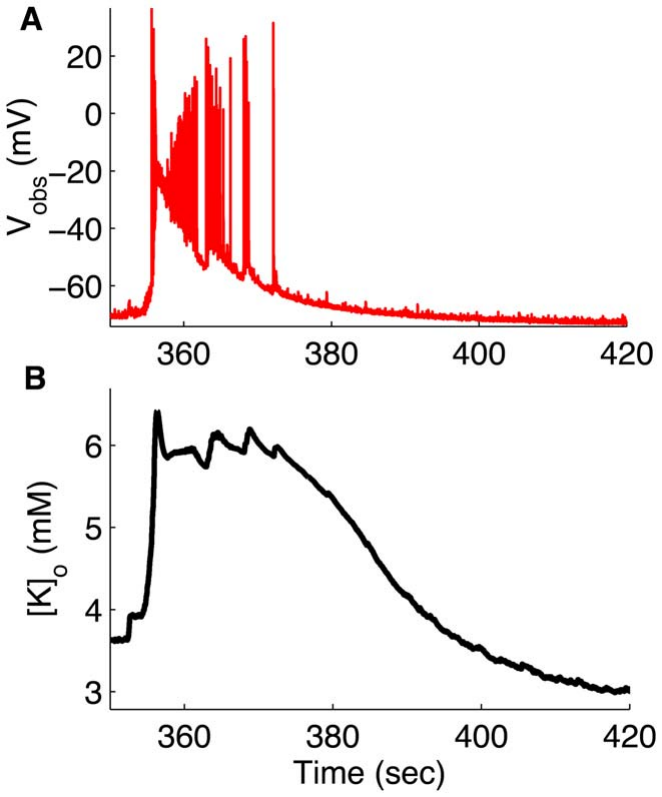
Expanded view of third seizure in **Figure 4** illustrating how 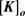 changes during a seizure. (A) membrane potential, 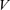, (B) extracellular potassium concentration, 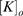.

In Figure 5, we show an expanded view of a single cell response during a single seizure from Figure 4. Extracellular potassium concentration increases several fold above baseline values during seizures [31]. During a single seizure, 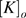 starts rising from a baseline value of 3.0mM as the seizure begins and peaks at 7mM at the middle of the seizure (Figure 5). Interestingly the 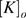 estimated by UKF matches very closely the measured 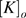 seen *in vitro* studies [34].

**Figure 6:**
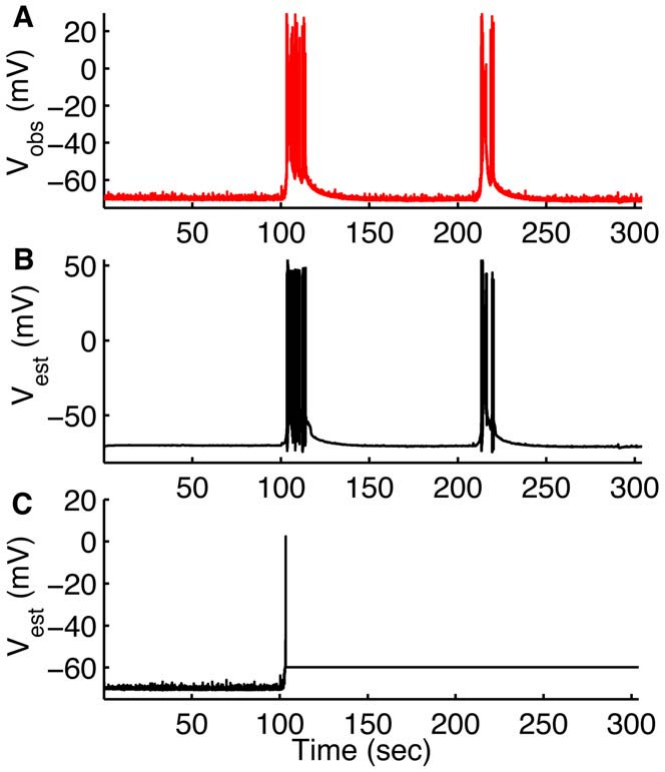
UKF cannot track seizures without microenvironmental 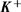 and 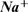 dynamics in the model. Observed (A) and estimated (B) membrane potential using the model with ion concentrations dynamics. In (C) we show estimated membrane potential using the model without ion concentrations dynamics.

Considering the slow time scale of seizure evolution (period of more than 100 seconds in our experiments), we test the importance of slow variables such as ion concentrations for seizure tracking. As shown in Figure 6, we found that including the dynamic intra- and extracellular ion concentrations in the model is necessary for accurate tracking of seizures. Using Hodgkin-Huxley type ionic currents with fixed intra- and extracellular ion concentration of 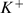 and 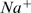 ions fails to track seizure dynamics in pyramidal cells (Figure 6C). We used physiologically normal concentrations of 4mM and 18mM for extracellular 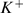 and intracellular 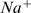 respectively for these simulations. The conclusion remains the same when higher 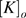 and 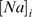 are used. A similar tracking failure is found while tracking the dynamics of OLM interneurons during seizures (not shown). To further emphasize the importance of ion concentrations dynamics for tracking seizures we calculate the Akaike's information criterion (AIC) for the two models used in Figure 6, i.e. the model with and without ion concentration dynamics. AIC is a measure of the goodness of fit of a model and offers a measure of the information lost when a given model is used to describe experimental observations. Loosely speaking, it describes the tradeoff between precision and complexity of the model [35]. We used equation (29) for the AIC measure. The AIC measure for the model without ion concentration dynamics is 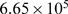. The model with ion concentration dynamics on the other hand has AIC value equal to 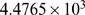, indicating the importance of ion concentration dynamics for tracking seizures.

**Figure 7:**
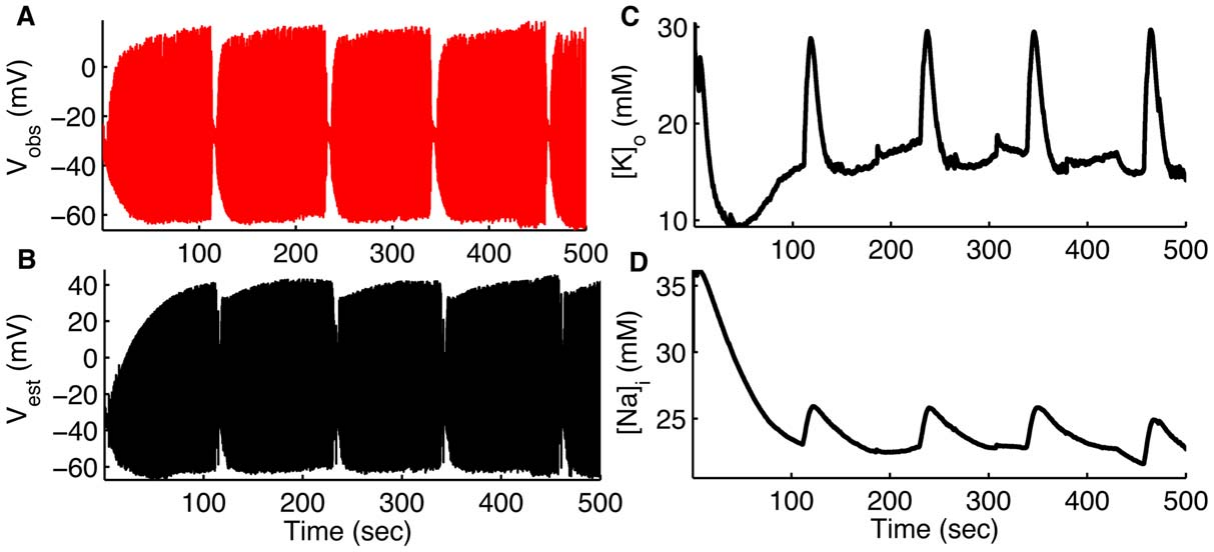
Assimilating seizure data from CA1 hippocampal OLM interneurons. Membrane potential measured (red) by whole cell recording from OLM interneurons during spontaneous seizures (A). In (B–D) we show membrane potential, 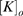, and 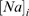 of the same cell respectively estimated (black) by using UKF. As shown in Figure S1, we also tracked the remaining variables for IN. Data provided by Jokubas Ziburkus. Panel (A) modified from [23] with permission American Physiological Society.

Pyramidal cells and interneurons in the hippocampus reside in different layers with different cell densities. To investigate whether there exist significant differences in the microenvironment surrounding these two cell types we assimilated membrane potential data from OLM interneurons in the hippocampus and reconstructed 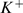 and 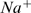 ion concentrations inside and outside the cells. As shown in Figure 7, both the baseline level and peak 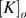 near the interneurons must be very high as compared to that seen for the pyramidal cells (cf. Figure 4B). This is an important prediction in light of the recently observed interplay between pyramidal cells and interneurons during *in vitro* seizures [23]; in these experiments pyramidal cells were silent when the interneurons were intensively firing. Following intense firing the interneurons entered a state of depolarization block simultaneously with the emergence of intense epileptiform firing in pyramidal cells. Such a novel pattern of interleaving neuronal activity is proposed to be a possible mechanism for the sudden drop in inhibition during seizures – it may be permissive of runaway excitatory activity. The mechanism leading to such interplay, specifically the reasons for differential firing patterns in pyramidal cells and interneurons are unknown. Our results here indicate the potential role of the neuronal microenvironment in producing such interplay. Our findings suggest that the 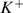 buffering mechanism in the OLM layer is weaker as compared with the pyramidal layer, thus causing higher 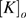 in the OLM layer. The higher 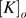 surrounding the interneurons causes increased excitability of the cell by raising the reversal potential for 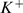 currents (higher than the pyramidal cells, see equation 7). The higher reversal potential for 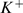 currents causes the interneuron to spontaneously burst fire at higher frequency and eventually drives the interneuron to transition into depolarization block when firing is peaked. As the INs enter the depolarized state, the inhibitory synaptic input from the INs to the PCs drops substantially, releasing PCs to generate the intense excitatory activity of seizures (equation 8, Figure S3). The collapse of inhibition due to the entrance of INs into a depolarized state also helps explain the sudden decrease in inhibition at seizure onset in neocortex described by Trevelyan, et al. [36] as the loss of *inhibitory veto*. As shown in Figure S1, we also tracked the remaining variables for the INs.

### Reconstructing Network Interaction

**Figure 8:**
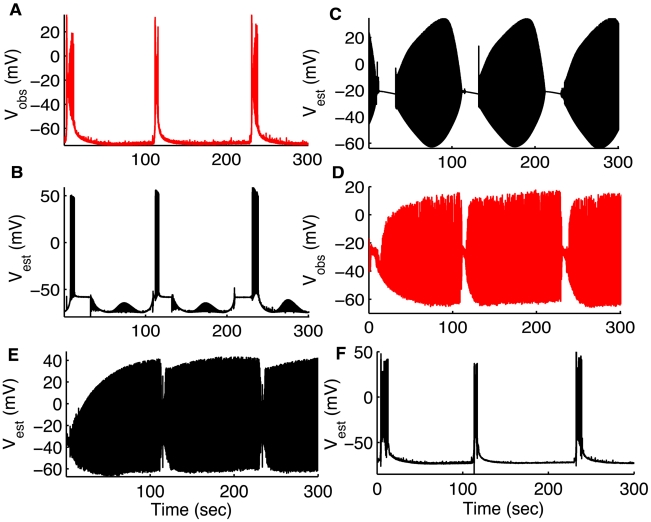
Reconstructing network interaction. Measured (A, red) and estimated (B, black) 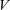 for pyramidal cell. (C) Estimated 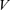 for interneuron. We used the membrane potential recorded from the pyramidal cell (shown in A, red) to not only reconstruct the full dynamics of the same pyramidal cell (only membrane potential shown in B, black) but also reconstructed the dynamics of the interneuron (only membrane potential shown in C, black). Simultaneously recorded 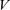 from the IN is shown in (D, red) for comparison. Estimates for intracellular 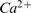 concentration and gating variables 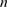, 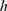 for PC are shown in Figure S2 and the synaptic variables, 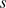, 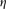 are shown in Figure S3. Estimated 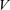 for IN (E) and PC (F) by assimilating measured 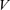 from IN (shown in (D)). (D–F) are converses of the simulations in (A–C). That is, In (D–F) we used membrane potential recorded from the interneuron (shown in D, red) to not only reconstruct the full dynamics of the same interneuron (only membrane potential shown in E, black) but also the coupled pyramidal cell (only membrane potential shown in F, black: compare with actual values shown in A, red). Simultaneous membrane potential measurements shown in (A,D) were from a pyramidal cell and OLM interneuron in the hippocampus using simultaneous dual whole cell patch clamp recordings demonstrating the firing interplay between these cells during *in vitro* seizures. Data provided by Jokubas Ziburkus. Panels (A,D) are modified from [23] with permission 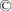 American Physiological Society.

Since the interaction of neurons determines network patterns of activity, it is within such interactions that we seek unifying principles for epilepsy. To demonstrate that the UKF framework can be utilized to study cellular interactions, we reconstructed the dynamics of one cell type by assimilating the measured data from another cell type in the network. In Figure 8 we only show the estimated membrane potentials, but we also reconstructed the remaining variables and parameters of both cells (Figures S2 and S3). We first assimilated the membrane potential of the PC to estimate the dynamics of the same cell and also the dynamics of a coupled IN (Figure 8A–D). Conversely, we estimate the dynamics of PC from the simultaneously measured membrane potential measurements of the IN (Figure 8D–F). As is evident from Figure 8 the filter framework is successful at reciprocally reconstructing and tracking the dynamics of these different cells within this network. In Figure S2, we show intracellular 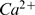 concentration and gating variables of 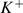 and 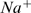 channels in PCs for simulation in Figure 8A–D. The variables modeling the synaptic inputs for both INs and PCs in Figure 8A–D are shown in Figure S3. As clear from Figure S3 (D), the variable 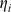 (equation 8) reaches very high values when the INs lock into depolarization block, shutting off the inhibitory inputs from INs to PCs.

## Discussion

There has been intense interest in the neuroscience communities in bringing control-theoretical tools to bear on neuronal encoding and decoding problems [37]–[45]. In all of this work, statistical models (continuous or point process) were fit to data recorded from neurons, and these empirical models incorporated into applications. Our use of control theoretic tools is very different. We built computational models from the physiological properties of neurons and their networks, as well as the properties of ion metabolism, *without data fitting*. Using these fundamental models of the physics of neuronal systems, we fuse these models with data – *data assimilation* – in a manner commonly applied in meteorology [1], [46]–[50]. We are aware of a recent laboratory demonstration in fluid mechanics using a simplified model of fluid dynamics (Boussinesq equations) in a similar manner as we have performed here [51] (see also [14]). Other authors have also recently discussed the importance and power of going beyond statistical empirical models in neuronal systems, and simulations have begun to explore the feasibility of carrying this out [52]–[54]. To our knowledge, our findings are the first experimental validation that a fundamental biophysical model of part of the brain can be employed to assimilate incomplete data and accurately reconstruct its network dynamics.

Our conjecture is that the parallels with numerical meteorology are deep. By the turn of the 20th century, it was apparent that the lack of periodicities in weather limited forecasts based on previous state (autoregressive) statistical models, and that integrating the actual equations of motion of the atmosphere would be required. Infeasible initially, the turning point came when integrating such models gave ‘first approximations that bore a recognizable resemblance to the actual motions’ [55]. Furthermore, the use of simplified dynamical models that retained the most important of the physical dynamics was a critical development [1].

Our findings suggest that an analogous use of biophysical models of neuronal processes using the recursive predictive strategies employed in meteorological data assimilation is now feasible. We are presently exploring such application in frameworks for model-based data assimilation and control of Parkinson's disease [15]. Experiments are underway exploring the application for seizures in the intact brain, and the assimilation of cognitive rhythms. The potential for such techniques to improve our understanding of the dynamics of single cells and neuronal networks is substantial.

### Conclusion

In conclusion, we have demonstrated the feasibility for data assimilation within neuronal networks using detailed biophysical models. In particular, we demonstrated that estimating the neuronal microenvironment and neuronal interactions can be performed by embedding our improving biophysical neuronal models within a model based state estimation framework. This approach can provide a more complete understanding of otherwise incompletely observed neuronal dynamics during normal and pathological brain function.

## Materials and Methods

### Model

**Figure 9:**
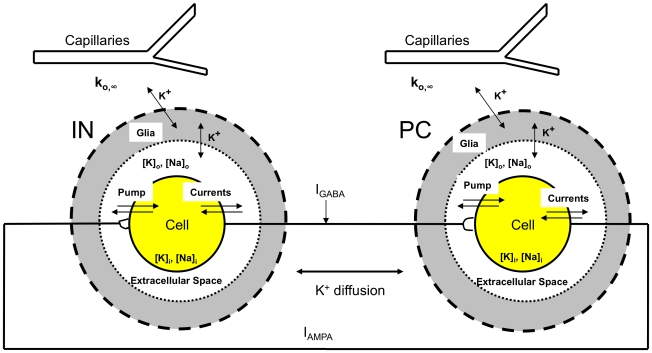
A schematic of the model dynamics. Potassium is released to the extracellular space and is pumped back to the cell by the ATP-dependent 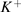
−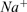 exchange pump, buffered by glia, and diffuses to the microenvironment where it interacts with capillaries. Sodium entering the cell through 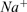 channels is pumped out of the cell by the ATP-dependent pump. Pyramidal cell (PC) and interneuron (IN) from the CA1 region of the hippocampus are coupled both synaptically and through lateral 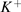 diffusion. Symbols used are defined in the text.

We used two-compartmental models for the pyramidal cells and interneurons: a cellular compartment and the surrounding extracellular microenvironment. The membrane potentials of both cells were modeled by Hodgkin-Huxley equations containing sodium, potassium, calcium-gated potassium (after-hyperpolarization), and leak currents. For the network model, the two cell types are coupled synaptically and through diffusion of potassium ions in the extracellular space. A schematic of the model is shown in Figure 9.

#### Membrane potential dynamics

The membrane potential 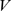 of the neurons is modeled with the following set of modified Hodgkin-Huxley equations [10], 
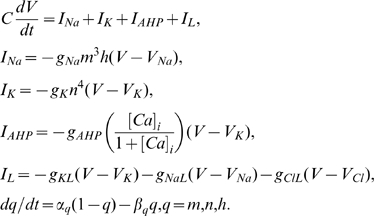
(1)where 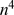 and 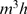 represent gating variables for potassium, 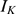, and sodium, 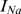, currents. The leak current, 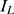, has three components: 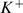 leak, 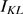, 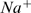 leak, 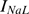, and chloride leak, 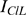. The after-hyperpolarization current 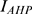 is only included in the pyramidal cell to account for its frequency adaptation. The meaning and values of parameters used in the model are given in Table 1.

**Table 1:** Model Parameters.

Parameter	Value	Description
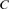	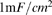	Membrane capacitance
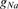	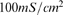	Conductance of Sodium Current
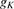	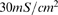	Conductance of potassium current
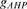	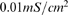	Conductance of afterhyperpolarization current
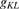	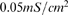	Conductance of potassium leak current
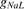	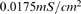	Conductance of sodium leak current
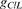	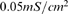	Conductance of chloride leak current
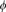	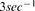	Time constant of gating variables
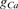	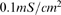	Conductance of calcium current
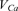	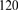 mV	Reversal potential of calcium
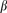	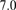	Ratio of intracellular to extracellular volume of the cell
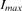	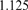 mM/sec	Maximum pump strength
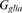	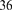 mM/sec	Maximum strength of glial uptake
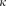	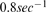	Diffusion constant of extracellular 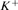
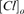	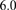 mM	Extracellular chloride concentration
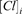	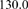 mM	Intracellular chloride concentration

Values and description of various parameters used in the model. All other parameters that are not given here are described in the “Materials and Methods” section.

The rate equations for the gating variables are
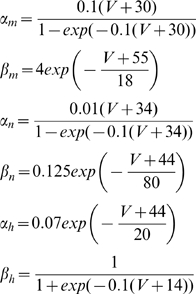
(2)


#### Ion concentrations dynamics

The current equations were augmented with dynamic variables representing the intra- and extracellular ion concentrations (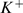, 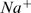, and 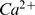). These ion concentrations are affected by the neuron's intrinsic ionic currents as well as a sodium-potassium pump current. The glial buffering, diffusion between the nearest neighbor cells, and diffusion into the environment of the cell (bath solution in slice preparation and vasculature *in vivo*) also modulate the potassium concentration in the microenvironmental extracellular space. The ion concentrations inside and outside the cell are coupled to the membrane voltage equations via the Nernst equation [10], [13], [25]. Finally, PCs and INs are connected to each other through synaptic inputs and diffusion of extracellular potassium between the nearest neighbor neurons.

Given the potassium ion currents 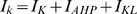, activity of the pump exchanging 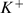 and 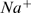, 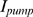, diffusion of potassium to the microenvironment, 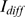, and glial buffering, 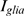, the extracellular potassium dynamics, 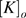, can be represented in the model as (Figure 9).
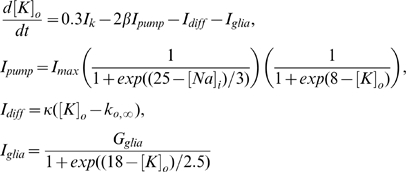
(3)where the 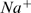
−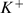 pump is modeled as a product of a sigmoidal functions, 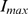 is the pump strength under normal conditions, and 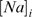 is the intracellular sodium concentration. Each sigmoidal term saturates for high values of internal sodium and external potassium respectively. More biophysically realistic models of pumps, such as those in [57] produce substantially similar results. 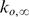 in the diffusion equation is the potassium concentration in the nearby reservoir. Physiologically, this would correspond to either the bath solution in a slice preparation, or the vasculature in the intact brain (noting that 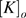 is kept below the plasma level by trans-endothelial transport). Both active and passive 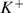 uptake into glia is incorporated into a simplified single sigmoidal response function that depends on extracellular 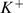 concentration with 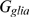 representing the maximum buffering strength. A similar but more physiological approach was used in [58]. Two factors allow the glia to provide a nearly insatiable buffer for the extracellular space. The first is the large size of the glial network. Second, the glial endfeet surround the pericapillary space, which, through interaction with arteriole walls, can effect blood flow; this in turn can increase the buffering capability of the glia [59]–[61].

We consider a spherical cell with a radius of 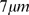. The diffusion coefficient of 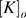 to the nearby reservoir 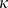, is obtained from Fick's law, 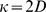/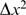, where 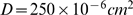/sec is the 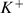 diffusion constant in neocortex [62], and 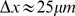 for brain reflects the average distance between capillaries [63]. The factor 0.3mM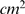/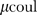
coul in equation (3) converts ionic current to concentration rate of change and is calculated using 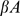/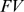 [10], where 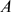, 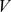 and 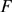 represent cell area, volume and Faraday constant respectively. 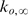 is equal to 3mM in physiological conditions, and the intra- to extracellular volume ratio 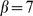 [64].

To complete the description of 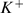 dynamics, we make the assumption that the flow of 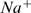 into the cell is compensated by flow of 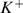 out of the cell to maintain bulk electroneutrality. Thus the internal potassium concentration (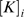) can be approximated by [10]

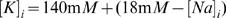
(4)where 140mM and 18mM respectively are the normal resting concentrations of 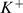 and 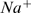 inside the cell.

The intracellular and extracellular 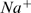 concentrations (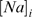, 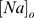) are also updated in the model as [10]

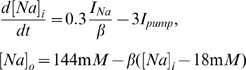
(5)where 144mM is the normal resting extracellular 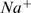 concentration. 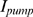 in equations (3) and (5) are multiplied by factor 2 and 3 respectively due to the fact that the 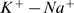 pump has an electrogenic 2∶3 ratio.

The intracellular 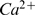 concentration, 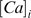, is modeled with the following rate equation [65]

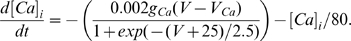
(6)


The reversal potentials for 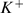, 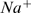 and 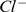 are updated based on the instantaneous ion concentrations using the Nernst equations
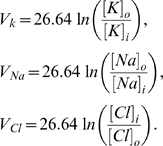
(7)


Equation (7) binds the ion concentrations dynamics to the Hodgkin-Huxley equations (1, 2).

#### Coupled cells model

The pyramidal cells and OLM interneurons are coupled both synaptically and through extracellular 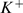 diffusion as shown in Figure 9. The following synaptic currents are added to the membrane potential equations [25]

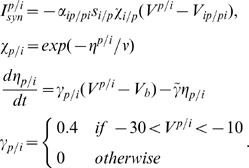
(8)


Where the superscripts 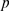 and 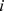 represent pyramidal cell and interneuron respectively. 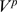 and 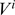 is the membrane potential of the PCs and INs respectively. The variable 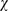 takes into account the firing interplay between pyramidal cells and interneurons [25]. Ziburkus, et al. [23] observed during *in vitro* seizures that pyramidal cells were silent when the interneurons were burst firing, followed by high frequency firing in pyramidal cells when interneurons were locked into a depolarized state called depolarization block. The variable 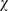 in equation (8) causes the synaptic input to drop to zero when the cells go to depolarization block. Various parameters used in equation (8) are: 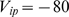, 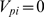, 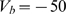, 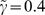, and 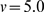. Synaptic strengths 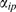, 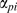 are mimicked according to 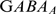 and AMPA inputs and values of 0.84 and 0.17, respectively, are used for the simulations. The variable 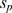 gives the temporal evolution of the synaptic input from the pyramidal cell to the interneuron and 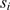 is the synaptic input from the interneuron to the pyramidal cell. 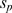 and 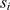 evolve as
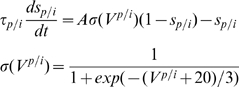
(9)The parameters 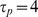 and 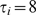 are the time constants for the excitatory and inhibitory synapses respectively and 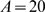.

In the case of coupled pyramidal cells and interneurons, the rate equation for 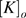 is updated by adding the following lateral diffusion term (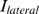)
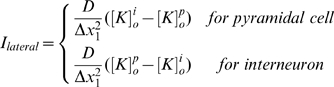
(10)where 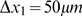 is the separation between the interneurons and pyramidal cells.

### Unscented Kalman Filter

To estimate and track the dynamics of the neuronal networks, we applied a nonlinear ensemble version of the Kalman filter, the unscented Kalman filter (UKF) [8], [9]. The UKF uses known nonlinear dynamical equations and observation functions along with noisy, partially observed data to continuously update a Gaussian approximation for the neuronal state and its uncertainty. At each integration step, perturbed system states that are consistent with the current state uncertainty, *sigma points*, are chosen. The UKF consists of integrating the system from the *sigma points*, estimating mean state values, and then updating the covariance matrix that approximates the state uncertainty. The Kalman gain matrix updates the new most likely state of the system based on the estimated measurements and the actual partially measured state. The estimated states (filtered states) are used to estimate the experimentally inaccessible parameters and variables by synchronizing the model equations to the estimated states. To estimate the system parameters from data, we introduced the unknown parameters as extra state variables with trivial dynamics. The UKF with random initial conditions for the parameters will converge to an optimal set of parameters, or in the case of varying parameters, will track them along with the state variables [11]–[13].

Given a function 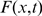 describing the dynamics of the system (equations 1–10 in our case), and an observation function 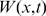 contaminated by uncertainty characterized in the covariance matrix 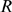, for a 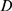-dimensional state vector with mean 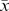 the UKF generates the 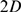 *sigma points* 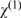, …, 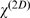 so that their sample mean and sample covariance are 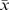 and 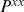. The *sigma points* are the 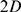 rows of the matrix
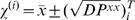
(11)The index 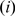 on the left-hand side corresponds to the 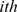 row taken from the matrix in the parenthesis on right-hand side. The square root sign denotes the matrix square root and 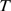 indicates transpose of the matrix. Sigma points can be envisioned as sample points at the boundaries of a covariance ellipsoid. In what follows, superscript tilde ( 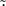 ) represents the *a priori* values of variables and parameter, i.e. the values at a given time-step 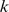 when observation up to time-step 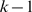 are available, while hat ( 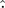 ) represents the *a posteriori* quantities, i.e. the values at time-step 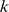 when observations up to time-step 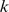 are available.

Applying one step of the dynamics 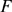 to the *sigma points* and calling the results 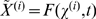, and denoting the observations of the new states by 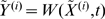, we define the means
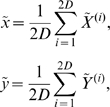
(12)where 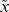 and 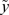 are the *a priori* state and measurement estimates, respectively. Now define the *a priori* covariances
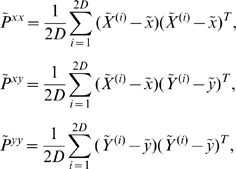
(13)of the ensemble members. The Kalman filter estimates of the new state and uncertainty are given by the *a posteriori* quantities
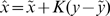
(14)and
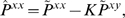
(15)where 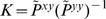 is the Kalman gain matrix and 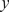 is the actual observation [3], [8], [9], [11]–[13]. Thus 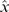 and 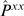 are the updated estimated state 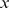 and covariance 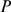 for the next step. The *a posteriori* estimate of the observation 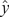 is recovered by 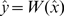. Thus by augmenting the observed state variables with unobserved state variables and system parameters, UKF can estimate and track both unobserved variables and system parameters.

#### Implementation of the UKF

In our simulations, the state 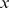 is the 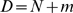 dimensional vector consisting of the 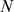 variable values (equations 1–10) describing the dynamics of neurons and the 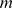 parameter values to be tracked. The one-step dynamics function 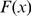 is the system of differential equations (equations 1–10). State vector 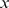 for a single PC is given as
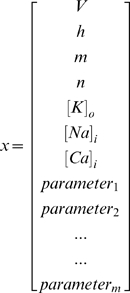
(16)Where 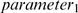, 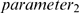, …. 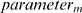 are the parameters that we want to track. For example, we tracked three parameters in Figure 4, replacing 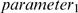, 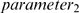, …. 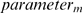 by 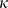, 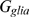, and 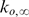 respectively in equation (16). For coupled PC and IN, the state vector 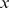 included variables 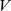, 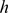, 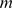, 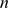, 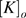, 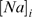, and 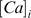 for IN along with four synaptic variables, 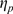, 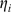, 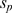, and 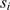 in order to represent the synaptic interactions between the two cells. The observation function 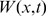 returned the first component of the vector 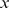 (membrane potential, 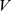) at given time t. We observed the membrane potential of the cell and treated the rest of the variables as unobserved. For most of our simulations we used an integration time-step dt = 0.01ms while the membrane potential of the neuron was measured each 0.1ms.

An iteration of the filter is performed in the following three steps (see [3], [8], [9] for more details).

*Initialization:* The filter is initialized as follows
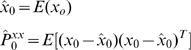
(17)where 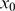 are the initial values of the state variables, and 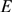 represent expectation.

*Prediction:* The following equations are used to propagate the state estimate and covariance from time-step (k−1) to k. First create a set of *sigma points* by applying equation (11) to system state equation (16)
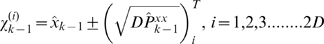
(18)The *sigma points* are transformed into vectors 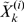 by using the nonlinear system of equations 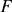 (1–10)
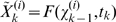
(19)The average of vectors 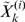 gives the *a priori* state estimate at time 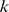.
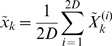
(20)The *a priori* error covariance is given as
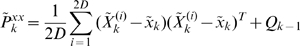
(21)where 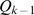 represents the process noise.

*Measurement Update:* We implemented the measurement update as follows. Given the current guess for the mean, 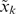, and covariance, 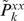 of 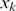, we choose new sigma points
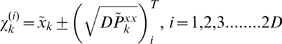
(22)This step can be omitted by using the *sigma points* from equation (18) to enhance the computational efficiency at the cost of performance [3]. The observation function 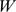 is used to transform the *sigma points* into predicted measurements, 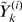 vector.
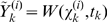
(23)The average of 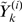 is the predicted measurement at time-step 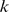:
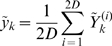
(24)Equations (23 and 24) are used to estimate the covariance of predicted measurement
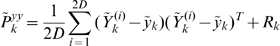
(25)where 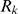 takes into account the measurement noise.

Next, we estimate the cross covariance between 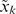 and 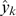


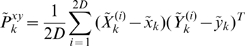
(26)Finally, the measurement at the time-step 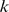 is used to update the state vector and its covariance
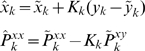
(27)where
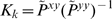
(28)


The *a posteriori* observation 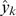 is recovered by 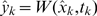.

We calculate the AIC measure for the two models used in Figure 6 using the following equations [35]

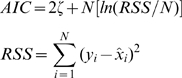
(29)Where 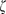 is the total number of parameters in the model, 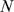 is the total number of data samples (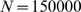 for examples in Figure 6), and 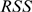 is the residual sum of squares. The model that includes ion concentration dynamics has four extra parameters, 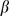, 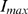, 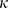, and 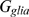. Therefore, we take 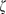 = 0 and 4 for the models without and with ion concentrations dynamics respectively.

All simulations were carried out using MATLAB on 2
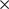
4 multi-core Mac Pro computer. The MATLAB code for the results is archived at ModelDB (http://senselab.med.yale.edu/modeldb/default.asp) and can also be provided by the authors upon request.

## Supporting Information

Figure S1 Estimates of remaining variables for the INs shown in Figure 7. (A) intracellular *Ca^2+^* concentration (arbitrary units), (B) *K^+^* channel gating variable, *n*, and (C) *Na^+^* channel gating variable, *h*.
(0.40 MB TIF)

Figure S2 Estimates of remaining variables for the PCs shown in Figure 8A, B. intracellular *Ca^2+^* concentration (arbitrary units) (A) and gating variables, *n* (B), *h* (C).
(0.30 MB TIF)

Figure S3 Estimates of synaptic variables for PCs and INs shown in Figure 8A–D. Synaptic variables, *s_p_* (A), *η_p_* (B), *s_i_* (C), and *η_i_* (D). As is clear from (D), *η_i_* reaches high values when the INs lock into depolarization block, causing *χ_i_* to approach zero thus shutting off the synaptic inputs from INs to PCs. When not in depolarization block, such as when fast spiking, *η_i_*→0 and *χ_i_*→0, not affecting synaptic currents.
(0.21 MB TIF)
