# Supplementary figures and images for: Assimilating Seizure Dynamics
Source: PLoS Comput Biol. 2010 May 6;6(5):e1000776. doi: 10.1371/journal.pcbi.1000776 (PMC2865517; doi:10.1371/journal.pcbi.1000776)

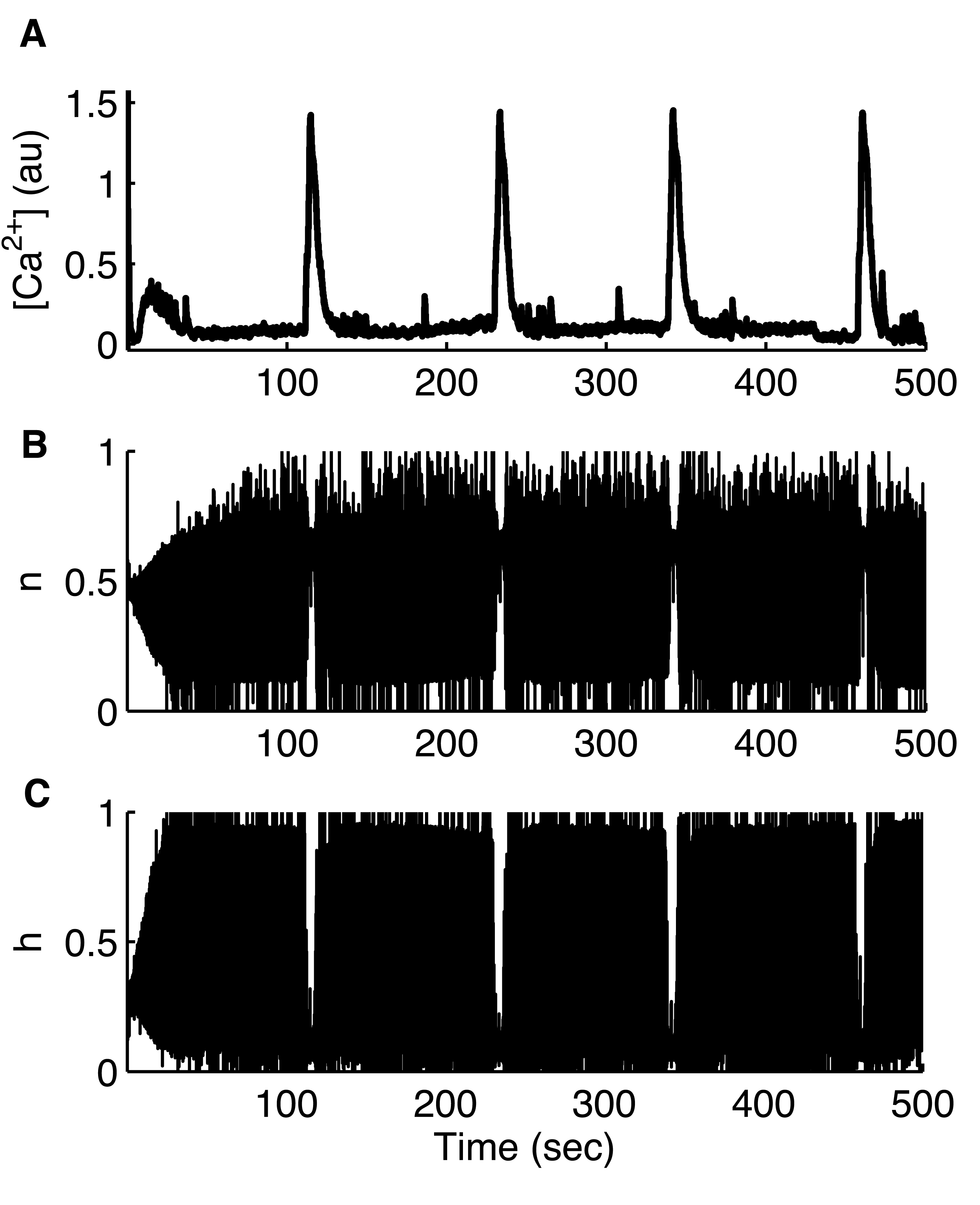

Supplement: Figure S1 — Estimates of remaining variables for the INs shown in Figure 7. (A) intracellular Ca2+ concentration (arbitrary units), (B) K+ channel gating variable, n, and (C) Na+ channel gating variable, h. (0.40 MB TIF) [file pcbi.1000776.s001.tif]

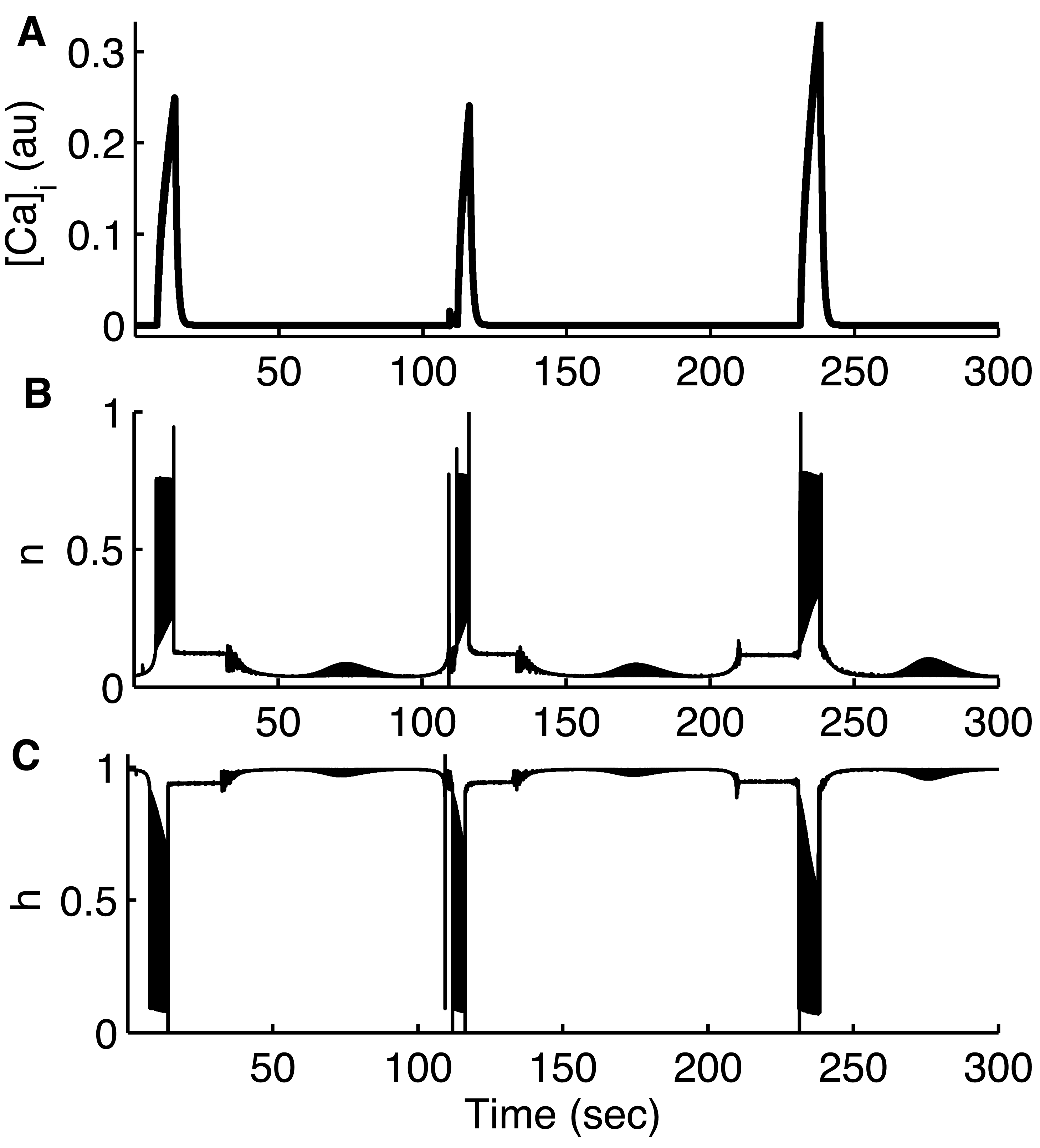

Supplement: Figure S2 — Estimates of remaining variables for the PCs shown in Figure 8A, B. intracellular Ca2+ concentration (arbitrary units) (A) and gating variables, n (B), h (C). (0.30 MB TIF) [file pcbi.1000776.s002.tif]

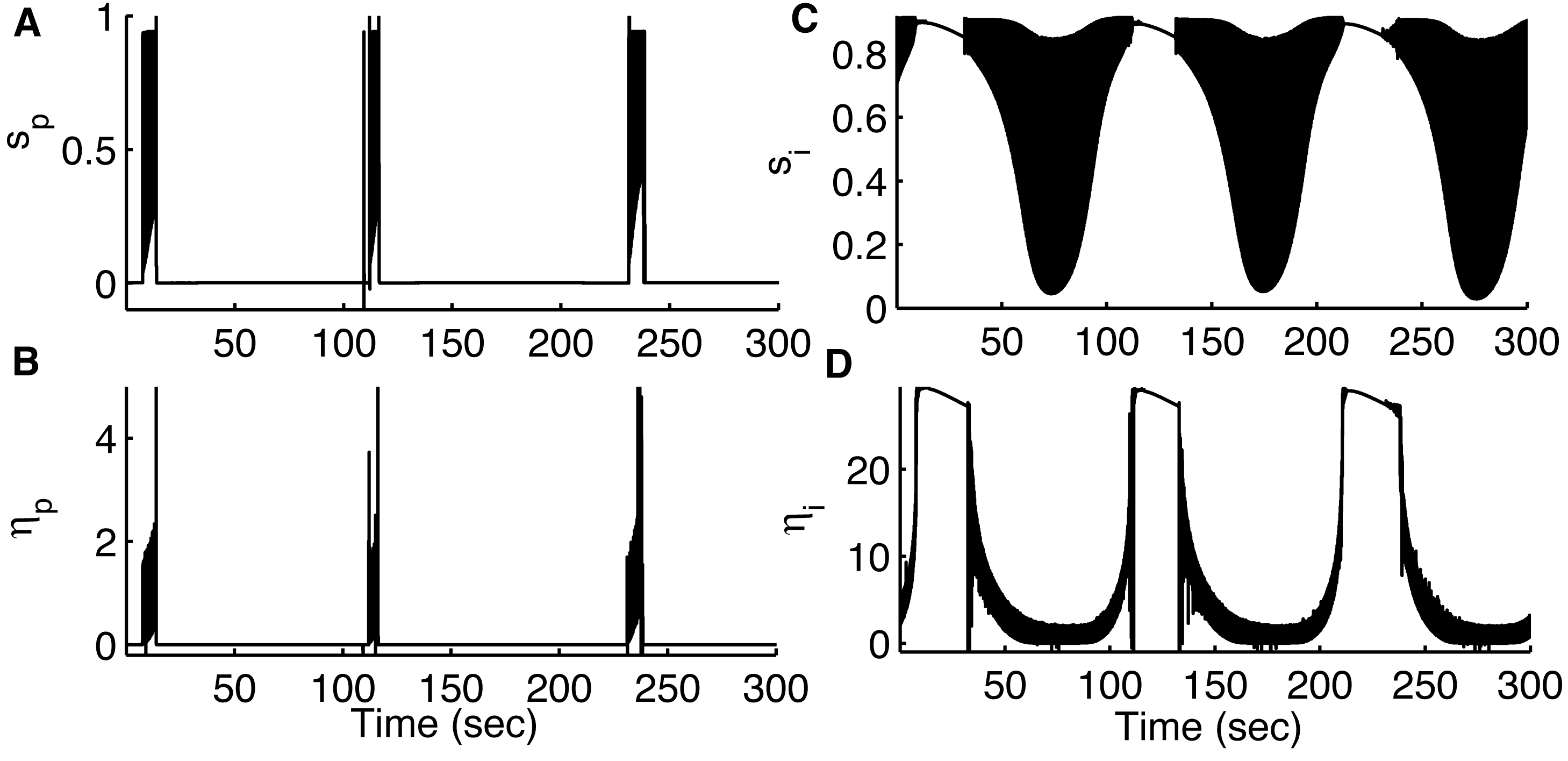

Supplement: Figure S3 — Estimates of synaptic variables for PCs and INs shown in Figure 8A–D. Synaptic variables, sp (A), ηp (B), si (C), and ηi (D). As is clear from (D), ηi reaches high values when the INs lock into depolarization block, causing χi to approach zero thus shutting off the synaptic inputs from INs to PCs. When not in depolarization block, such as when fast spiking, ηi→0 and χi→0, not affecting synaptic currents. (0.21 MB TIF) [file pcbi.1000776.s003.tif]
